# Comment on "Effect of exposure to ambient PM_2.5_ pollution on the risk of respiratory tract diseases: a meta-analysis of cohort studies"

**DOI:** 10.7555/JBR.36.20220091

**Published:** 2022-05-28

**Authors:** Ammanie Abdul-Fatah, Jia Lu Gao, David M. Stieb

**Affiliations:** 1 Department of Health Sciences, Carleton University, Ottawa, Ontario K1S 5B6, Canada; 2 Department of Population Medicine, University of Guelph, Guelph, Ontario N1G 2W1, Canada; 3 Environmental Health Science and Research Bureau, Health Canada, Ottawa, Ontario K1A 0K9, Canada; 4 School of Epidemiology and Public Health, University of Ottawa, Ottawa, Ontario K1G 5Z3, Canada

Dear Editor,

We reviewed the paper by Liu *et al*^[1]^, as part of an umbrella review on PM_2.5_ and the incidence of respiratory outcomes. We determined that the authors did not account for the PM_2.5_ increments based upon which primary studies reported their results. For instance, Young *et al*^[2]^ reported an odds ratio (OR) of 1.20 (95% confidence interval [CI]: 0.99, 1.46) per 3.6 µg/m^3^ PM_2.5_ for incident asthma. Likewise, Bennett *et al*^[3]^ reported an OR of 1.08 (95% CI: 0.79, 1.48) per 1 μg/m^3^ PM_2.5_ for incident wheezing. Liu *et al*^[1]^ simply pooled these and other results "as is", ignoring the differing PM_2.5_ increments, meaning that their pooled estimates are of limited value.

Results from primary studies should be standardized to the same pollutant increment prior to pooling. In our opinion, this could have been detected at the time of peer review, since the authors did not report the PM_2.5_ increment upon which their pooled estimates were based. While we did not examine their findings for other outcomes, we suspect that the same error applies.

We have identified this issue in other recent systematic reviews, including Pranata *et al*^[4]^, Zhao *et al*^[5]^, Han *et al*^[6]^, and Zhang *et al*^[7]^.

Yours Sincerely,Ammanie Abdul-Fatah^1,3^, Jia Lu Gao^2,3^, David M. Stieb^3,4,✉^ ^1^Department of Health Sciences,Carleton University, Ottawa, Ontario K1S 5B6, Canada;^2^Department of Population Medicine,University of Guelph,Guelph, Ontario N1G 2W1,Canada;^3^Environmental Health Science and Research Bureau, Health Canada,Ottawa, Ontario K1A 0K9,Canada;^4^School of Epidemiology and Public Health,University of Ottawa,Ottawa, Ontario K1G 5Z3,Canada.^✉^Tel/Fax:+1-604-666-3701/+1-604666-5741, E-mail: dave.stieb@hc-sc.gc.ca.
